# Clinical relevance of serum uric acid and abdominal aortic‐calcification in a national survey

**DOI:** 10.1002/clc.23433

**Published:** 2020-07-28

**Authors:** Yen‐Wei Li, Wei‐Liang Chen

**Affiliations:** ^1^ Department of Psychiatry Tri‐Service General Hospital; and School of Medicine, National Defense Medical Center Taipei Taiwan, Republic of China; ^2^ Division of Geriatric Medicine, Division of Family Medicine, Department of Family and Community Medicine Tri‐Service General Hospital; and School of Medicine, National Defense Medical Center Taipei Taiwan, Republic of China

**Keywords:** arteriosclerosis, cardiovascular disease, hyperuricemia, vascular calcification

## Abstract

**Background:**

Hyperuricemia was often found in subjects with an elevated risk of cardiovascular disease (CVD). Abdominal aortic‐calcification (AAC) is significantly associated with subclinical atherosclerotic disease.

**Hypothesis:**

The aim of this study is to evaluate the relationship between serum uric acid (SUA) level and AAC from a national database.

**Methods:**

A total of 2765 eligible participants older than 40 years who received dual‐energy X‐ray absorptiometry (DXA) scans with SUA data were enrolled from the National Health and Nutrition Examination Survey (2013‐2014). The association between SUA level and AAC was analyzed using multivariate regression models for covariate adjustment.

**Results:**

After categorizing SUA level into four quartiles, the odds ratios for the presence of subclinical atherosclerosis via contrasting the highest SUA quartile with the lowest SUA quartile were 1.876 (95% CI = 1.298‐2.711), 2.038 (95% CI = 1.303‐3.187), 1.935 (95% CI = 1.221‐3.065), and 1.956 (95% CI = 1.225‐3.124) (all, *P* value less than .05) in Model 1, Model 2, Model 3, and Model 4, respectively. The above relationship remained still in the fully adjusted model for the male but not female subjects. The optimal SUA cutoff value was 6.35 mg/dL for male and 5.25 mg/dL for female to predict the presence of subclinical atherosclerosis.

**Conclusions:**

Our results explore the promising evidences that SUA level showed a positive correlation with AAC score in a dose‐response manner. These findings decisively indicated that SUA may act as a promising tool to forecast the incidence of subclinical atherosclerosis in males.

**Abbreviations**
AAC: abdominal aortic‐calcification
AUROC: area under the receiver operating characteristic
BMI: body mass index
CREA: serum creatinine
CVD: cardiovascular disease
DXA: dual‐energy X‐ray absorptiometry
LDL‐C: low‐density lipoprotein cholesterol
ROC: receiver operating characteristic
SUA: serum uric acid

## 1 INTRODUCTION

As the leading cause of mortality since the early 20th century in the developed world, cardiovascular disease (CVD) has exceeded infectious disease and become one of the major threats to human health nowadays.^1^ Atherosclerosis, among the wide array of problems that may arise within the cardiovascular system, has always been the underlying cause of about 50% of all deaths in westernized society.^2^ For the purpose of evaluating the individualized cardiovascular risk, it's essential to recognize atherosclerosis in subclinical stages.

As the end product of purine catabolic metabolism, uric acid, which level in serum is influenced by multiple factors, including exogenous ingestion (particularly with an animal protein‐rich diet), endogenous production by the liver, and renal excretion.^3^ Serum uric acid (SUA) is an independent risk factor for CVD events including stroke, congestive heart failure, coronary heart disease, and even cardiovascular mortality in an elevated risk or general population.^4^ Previous study by TavIl Y et al demonstrated that raised SUA level has been shown to be associated with atherogenesis, independent from hypertension.^5^

Abdominal aortic‐calcification (AAC), measured via AAC score described by Kauppila LI et al in 1997^6^ or another semiquantitative method is significantly associated with subclinical atherosclerotic disease and an independent predictor of subsequent vascular morbidity and mortality.^7^

To our knowledge, no nationally representative US studies on the relationship between SUA and AAC are available. Hence, we investigated the association between SUA and AAC using results from the National Health and Nutrition Examination Survey 2013‐2014 (NHANES 2013‐2014).

## 2 METHODS AND MATERIALS

### 2.1 Study populations

**FIGURE 1:**
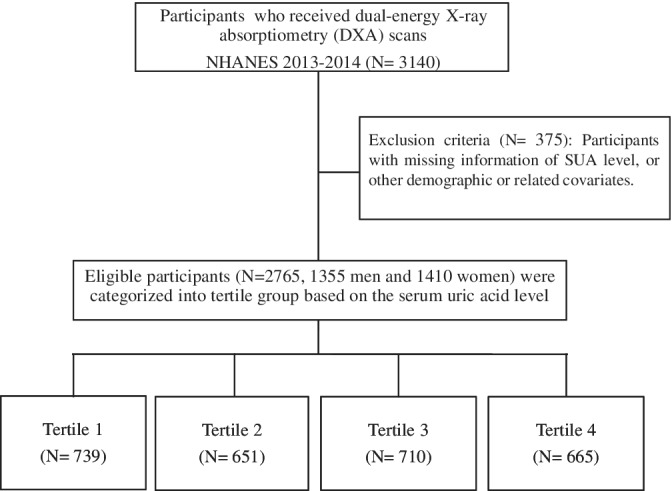
Flow diagram of our study. SUA, serum uric acid

Data were derived from the 2013‐2014 NHANES, a national survey designed by the National Center for Health Statistics of the Centers for Diseases Control and Prevention using a stratified, multistage, clustered probability sample design. The survey contained a large‐scale household interview (information on medical history, ethnicity/race, age, and gender) and a sequential physical examination at a particularly equipped Mobile Examination Center (MEC). In this cross‐sectional study we included participants over the age of 40 who received dual‐energy X‐ray absorptiometry (DXA) scans (n = 3140). We excluded subjects with lost information of SUA level, or other demographic or associated covariates. The final analytical sample of 2765 participants was composed of 1355 men and 1410 women who were categorized into tertile group based on the SUA level (Figure 1). Ethics approval was approved by the National Center for Health Statistics Ethics Review Board. All participants had signed an informed consent before study participation.

### 2.2 AAC score

As an acknowledged prognosticator of cardiovascular mortality,^7^ the evaluation of AAC via lateral lumbar spine (vertebrae L1‐L4) scans was acquired on Hologic Discovery model A densitometers (Hologic, Inc., Marlborough, Massachusetts) and quantified by the Kauppila score system and Schousboe score system^8^ which defines the classification of different AAC scoring techniques shown in Supplementary Table 1. Briefly, the abdominal aorta was divided into four parts immediately anterior to each of the lumbar vertebrae L1‐L4. For each part, aortic‐calcification severity in the anterior and posterior longitudinal walls was graded separately on a 0‐3 scale. The calcium lesions are graded as follows: “0” represents no calcification; “1” represents calcification length less than 1/3 of vertebra; “2” represents the calcification length extended from 1/3 to 2/3 of vertebra; “3” represents calcification length more than 2/3 of vertebra. With this numerical grading, the score could vary from a minimum of 0 to a maximum of 24 points to form AAC‐24 score.^9^ On the other hand, ranging from “0” to “4” and the total score range is 0 to 8, AAC‐8 score evaluates the entire length of calcification of the anterior and posterior aortic walls in front of L1 to L4. One grade is given for the presence of calcification in the abdominal aorta alongside one vertebra on either the anterior or posterior side. If the calcification has an aggregate length of more than one vertebra, the grade increases one point and so forth.^8^

Subjects were ineligible for DXA examination in this research if they were younger than 40 years old, pregnant, weighed more than 450 pounds (DXA table limitation) or used radiographic contrast material in past 7 days. AAC Total 24 Score above the 75th percentile (2 points) was defined as subclinical atherosclerosis in the present study. While AAC total 24 score > 6 has been thought of as noteworthy calcification and used as a cutoff point in prior research.^7^

### 2.3 Biochemical measurements

Peripheral blood was commenced from all subjects in the morning after an overnight fast about 12 to 14 hours to obtain biochemical analysis. Blood samples were collected in ethylenediaminetetraacetic acid (EDTA) containing tubes via a venous puncture for all subjects at the same room. Fasting glucose was measured in hexokinase (HK) method (Cobas Integra 400 plus, Roche Instrument Center, Rotkreuz, Switzerland). Concentration of serum low‐density lipoprotein cholesterol (LDL‐C) had been analyzed by means of an enzymatic cholesterol assay following dextran sulfate precipitation. Serum creatinine (CREA) was determined enzymatically on a Cobas 6000 (Roche). Level of SUA was measured with an uricase‐based method using the Hitachi 7150 automatic biochemical analyzer (Hitachi, Tokyo, Japan).

### 2.4 Assessment of other covariates

In Supplementary Table 2, we demonstrated a correlation table with all critical variables based on previous research. The associated information concerning variables including ethnicity/race (Mexican American, other Hispanic, non‐Hispanic white, and non‐Hispanic black), sex, age, body mass index (BMI), smoking, and medical conditions diagnosed by doctors, including angina/angina pectoris, stroke, and congestive heart failure, were collected through self‐report. BMI was determined according to the formula that divided the subject's weight in kilograms by the square of the height in meters (kg/m^2^). Smoking status was assessed with the item, “Smoked at least 100 cigarettes in life.” Other medical history like congestive heart failure was classified through a query like “Has a doctor or health professional ever told you that you had congestive heart failure (or angina/angina pectoris, or stroke)?”

### 2.5 Statistical analyses

All statistical analyses were performed using SPSS (Version 22.0 for Windows, SPSS, Inc., Chicago, Illinois). Discrete variables are demonstrated as frequency counts and percentages, while continuous variables are demonstrated as the mean and SD. The Student *t* test was applied to continuous data, and the *χ* ^2^ test was applied to discrete data. A two‐sided *P* < .05 was thought of as statistical significance. A receiver‐operator characteristic (ROC) curve was plotted to analyze the discriminative power of the prediction tools, and the area under the ROC (AUROC) and the corresponding 95% confidence intervals (CI) were calculated. We performed a quartile‐based analysis in this research via categorizing SUA level into four quartiles. The cutoff values for SUA level were as the following: Q1 < 4.5 (mg/dL), 4.5 < Q2 < 5.3 (mg/dL), 5.3 < Q3 < 6.3 (mg/dL), and 6.3 < Q4 (mg/dL). The correlation between SUA level and AAC was evaluated by the following extended‐model linear regression: Model1 = unadjusted; Model2 was adjusted for race, age, BMI, and gender; Model 3 = Model 2 + LDL‐cholesterol, fasting plasma glucose, and serum creatinine; Model 4 = Model 3 + history of angina/angina pectoris, stroke, smoking, and congestive heart failure.

## 3 RESULTS

### 3.1 Baseline characteristics of total participants according to SUA quartile

Table 1 revealed the demographic characteristics of the 2765 enrolled subjects stratified by SUA quartiles. The mean age was 58.54 ± 12.00 years and the mean SUA level was 5.44 ± 1.38 mg/dL. The participants in the highest quartile was predominated by men and more probably to be older, have more history of congestive heart failure, higher BMI, and higher CREA level (all, *P* < .05).

**TABLE 1:** Demographic and characteristics of enrolled participants and their association between SUA levels which were divided into four groups

Variables	Q1 SUA (<267.66) (n = 739)	Q2 SUA (267.66‐ < 315.24) (n = 651)	Q3 SUA (315.24‐ < 374.72) (n = 710)	Q4 SUA (374.72‐ < 666.18) (n = 665)	Total (41.64‐666.18) (n = 2765)	*P* value
Continuous variables, (median [IQR])						
Age (years)	55.00 (19.00)	57.00 (20.00)	59.50 (20.00)	62.00 (20.00)	58.00 (20.00)	<.001
BMI (kg/m^2^)	26.00 (6.90)	27.20 (6.45)	27.75 (6.30)	28.50 (6.30)	27.50 (6.80)	<.001
LDL‐C(mmol/L)	2.87 (1.14)	2.93 (1.37)	2.93 (1.22)	2.93 (1.30)	2.93 (1.24)	.334
FPG (mmol/L)	5.22 (0.83)	5.38 (1.00)	5.49 (1.11)	5.66 (1.11)	5.44 (1.05)	.247
CREA (μmol/L)	64.53 (17.68)	71.60 (23.87)	82.21 (24.75)	89.28 (28.29)	76.02 (26.52)	<.001
SUA (μmol/L)	231.97 (41.64)	291.45 (23.79)	344.98 (23.79)	422.31 (59.48)	321.19 (107.06)	<.001
Categorical variables, (%)						
Male	178 (24.1)	262 (40.2)	444 (62.5)	471 (70.8)	1355(49.0)	<.001
Non‐Hispanic white	338 (45.7)	292 (44.9)	317 (44.6)	282 (42.4)	1229(44.4)	<.001
CHF history	18 (2.4)	13 (2.0)	21 (3.0)	44 (6.6)	96(3.5)	<.001
Angina/Angina pectoris history	19 (2.6)	18 (2.8)	25 (3.5)	26 (3.9)	88(3.2)	.540
Stroke history	26 (3.5)	32 (4.9)	29 (4.1)	26 (3.9)	113(4.1)	.827
Smoking history	317 (42.9)	291 (44.7)	333 (46.9)	335 (50.4)	1276(46.1)	.090

*Note:* CHF history, ever told had congestive heart failure (MCQ160b). Smoking history, smoked at least 100 cigarettes in life (SMQ020).

Abbreviations: BMI, body mass index; CREA, serum creatinine; FPG, fasting plasma glucose; LDL‐C, low‐density lipoprotein cholesterol; SUA, serum uric acid.

### 3.2 Association between SUA and AAC scores

The regression analysis between SUA level and different types of AAC scores categorized by gender are shown in Table 2. In the total participants, SUA level was significant associated with the AAC total 24 score in all models. The above association remained still in Model 3 and Model 4, which was fully adjusted, for the male participants; while only to do so in Model 1 for the female participants. The relationship between SUA level and AAC posterior 8 Score was significant in all models for the entire and male participants; but fail to persist in fully adjusted model for females. In addition, significant correlation between SUA level and AAC total 8 score was demonstrated in the total participants as well; whereas the aforesaid correlation only remained significant in Model 3 and Model 4 for the male but not female subjects. After stratification of SUA level into four quartiles, the β coefficients of AAC total 24 score, AAC posterior 8 score, and AAC total 8 score in participants with Q4SUA were significantly different with Q1SUA in the entire 4 models. (Table 3, all *P* < .05, p for trend<0.01).

**TABLE 2:** Association between SUA (as continuous variables) and AAC scores (as continuous variables) categorized by gender

Variable		Model 1		Model 2		Model 3		Model 4	
		β (95% CI)	*P* value	β (95% CI)	*P* value	β (95% CI)	*P* value	β (95% CI)	*P* value
SUA	AAC total 24 score								
	Total	0.043 (−0.029, 0.077)	.377	0.027 (−0.043, 0.073)	.607	0.038 (−0.037, 0.079)	.477	0.033 (−0.039, 0.076)	.530
	Male	0.058 (−0.052, 0.381)	.137	0.073 (0.000, 0.413)	.050	0.076 (0.004, 0.421)	.045	0.080 (0.018, 0.430)	.033
	Female	0.114 (0.106, 0.509)	.003	0.065 (−0.028, 0.380)	.090	0.032 (−0.123, 0.296)	.416	0.011 (−0.174, 0.236)	.766
	AAC anterior 8 score								
	Total	0.054 (0.000, 0.047)	.047	0.047 (−0.005, 0.046)	.109	0.051 (−0.003, 0.047)	.088	0.050 (−0.003, 0.047)	.099
	Male	0.015 (−0.029, 0.043)	.701	0.036 (−0.019, 0.052)	.349	0.045 (−0.015, 0.057)	.250	0.045 (−0.014, 0.057)	.237
	Female	0.093 (0.009, 0.078)	.015	0.047 (−0.014, 0057)	.231	0.014 (−0.030, 0.043)	.725	−0,004 (−0.039, 0.035)	.914
	AAC posterior 8 score								
	Total	0.101 (0.026, 0.083)	<.001	0.095 (0.020, 0.082)	.001	0.090 (0.017, 0.080)	.002	0.089 (0.017, 0.079)	.002
	Male	0.080 (0.002, 0.091)	0.042	0.098 (0.014, 0.100)	.010	0.097 (0.013, 0.100)	.011	0.102 (0.016, 0.102)	.007
	Female	0.120 (0.027, 0.115)	0.002	0.077 (0.001, 0.091)	.047	0.046 (−0.019, 0.074)	.254	0.027 (−0.029, 0.062)	.488
	AAC total 8 score								
	Total	0.084 (0.028, 0.126)	.002	0.078 (0.019, 0.124)	.008	0.076 (0.017, 0.123)	.010	0.075 (0.017, 0.121)	.009
	Male	0.054 (−0.023, 0.130)	.172	0.074 (0.000, 0.148)	.050	0.077 (0.003, 0.152)	.043	0.081 (0.007, 0.155)	.032
	Female	0.113 (0.039, 0.188)	.003	0.067 (−0.009, 0.142)	.084	0.033 (−0.045, 0.110)	.408	0.013 (−0.063, 0.090)	.735

*Note:* Model 1: unadjusted. Model 2: adjusted by (race, age, BMI, gender). Model 3: adjusted by Model 2 + (LDL‐ cholesterol, fasting plasma glucose, serum creatinine). Model 4: adjusted by Model 3 + (angina/angina pectoris history, stroke history, smoking history, congestive heart failure history).

Abbreviation: AAC, abdominal aortic‐calcification; CI, confidence intervals; SUA, serum uric acid.

**TABLE 3:** Association between SUA (as quartiles comparison) and AAC scores (as continuous variables)

SUA quartiles	Model 1		Model 2		Model 3		Model 4	
	β (95% CI)	*P* value	β (95% CI)	*P* value	β (95% CI)	*P* value	β (95% CI)	*P* value
AAC total 24 Score								
Q2 vs Q1	0.018 (−0.390, 0.687)	.589	0.019 (−0.349, 0.662)	.544	0.022 (−0.318, 0.690)	.469	0.021 (−0.315, 0.673)	.478
Q3 vs Q1	0.056 (−0.078, 0.953	.096	0.051 (−0.112, 0.921)	.125	0.055 (−0.079, 0.952)	.097	0.059 (−0.044, 0.968)	.073
Q4 vs Q1	0.108 (0.354, 1.413)	.001	0.096 (0.230, 1.339)	.006	0.096 (0.225, 1.340)	.006	0.098 (0.256, 1.349)	.004
P for trend	0.093 (0.124, 0.459)	.001	0.083 (0.082, 0.438)	.004	0.083 (0.081, 0.439)	.005	0.086 (0.093, 0.444)	.003
AAC anterior 8 score								
Q2 vs Q1	0.017 (−0.068, 0.116)	.610	0.022 (−0.056, 0.120)	.477	0.026 (−0.050, 0.125)	.405	0.026 (−0.050, 0.123)	.406
Q3 vs Q1	0.055 (−0.015, 0.161)	.103	0.060 (−0.009, 0.170)	.079	0.066 (−0.001, 0.178)	.053	0.069 (0.004, 0.181)	.041
Q4 vs Q1	0.088 (0.032, 0.212)	.008	0.089 (0.027, 0.220)	.012	0.093 (0.033, 0.226)	.009	0.095 (0.036, 0.228)	.007
P for trend	0.077 (0.013, 0.070)	.005	0.079 (0.011, 0.073)	.008	0.082 (0.013, 0.075)	.006	0.085 (0.014, 0.076)	.004
AAC posterior 8 score								
Q2 vs Q1	0.025 (−0.069, 0.160)	.438	0.027 (−0.062, 0.157)	.393	0.027 (−0.060, 0.157)	.382	0.026 (−0.060, 0.153)	.390
Q3 vs Q1	0.057 (−0.015, 0.204)	.090	0.053 (−0.022, 0.201)	.117	0.054 (−0.021, 0.201)	.112	0.057 (−0.014, 0.204)	.088
Q4 vs Q1	0.110 (0.078, 0.303)	.001	0.100 (0.053, 0.293)	.005	0.094 (0.042, 0.283)	.008	0.096 (0.049, 0.285)	.005
P for trend	0.093 (0.026, 0.097)	.001	0.084 (0.018, 0.095)	.004	0.079 (0.014, 0.092)	.007	0.083 (0.017, 0.093)	.004
AAC total 8 Score								
Q2 vs Q1	0.022 (−0.129, 0.261)	.506	0.025 (−0.108, 0.261)	.415	0.028 (−0.101, 0.267)	.375	0.027 (−0.100, 0.262)	.380
Q3 vs Q1	0.058 (−0.022, 0.351)	.083	0.059 (−0.021, 0.356)	.082	0.062 (−0.012, 0.365)	.066	0.065 (0.001, 0.370)	.049
Q4 vs Q1	0.105 (0.118, 0.501)	.002	0.100 (0.092, 0.497)	.004	0.098 (0.086, 0.493)	.005	0.101 (0.097, 0.497)	.004
P for trend	0.090 (0.041, 0,163)	.001	0.086 (0.032, 0.162)	.003	0.085 (0.031, 0.162)	.004	0.088 (0.035, 0.164)	.002

*Note:* Model 1: unadjusted. Model 2: adjusted by (race, age, BMI, gender). Model 3: adjusted by Model 2 + (LDL‐ cholesterol, fasting plasma glucose, serum creatinine). Model 4: adjusted by Model 3 + (angina/angina pectoris history, stroke history, smoking history, congestive heart failure history).

Abbreviation: AAC, abdominal aortic‐calcification; CI, confidence intervals; SUA, serum uric acid.

### 3.3 Odds ratios for presence of subclinical atherosclerosis according to quartiles comparison of SUA

Supplementary Table 3 presented the odds ratios (OR) of subclinical atherosclerosis at different quartiles comparison of SUA. Compared with the participants with Q1SUA, those with Q4SUA had greater odds for subclinical atherosclerosis in all four models (Model 1: OR 1.876, 95% CI 1.298‐2.711; Model 2: OR 2.038, 95% CI 1.303‐3.187; Model 3: OR 1.935, 95% CI 1.221‐3.065; Model 4: OR 1.956, 95% CI 1.225‐3.124). The above relationship remained still in the fully adjusted model for the male but not female subjects. These findings decisively indicated that SUA may act as a promising tool to forecast the incidence of subclinical atherosclerosis in males.

### 3.4 SUA cutoff values for predicting the presence of subclinical atherosclerosis

Optimal SUA cutoff values for predicting the presence of subclinical atherosclerosis were determined using Youden's index derived by ROC analyses in different gender groups. (Supplementary Table 4) The optimal SUA cutoff value was 6.35 mg/dL for male and 5.25 mg/dL for female. AUROC estimate for female (0.577; 95% CI, 0.539‐0.616) was higher than that for male (0.516; 95% CI, 0.478‐0.555). Based on the ROC analyses, we examine the relationship between SUA cutoff value and subclinical atherosclerosis (Table 4). The aforesaid relationship, consistent with Supplementary Table 3, remained significant in the fully adjusted model for the male but not female subjects.

**TABLE 4:** Association between SUA cutoff value and subclinical atherosclerosis categorized by gender

Gender	Serum uric acid cutoff values	Model 1		Model 2		Model 3		Model 4	
		OR (95% CI)	*P* value	OR (95% CI)	*P* value	OR (95% CI)	*P* value	OR (95% CI)	*P* value
Male	6.35 mg/dL	1.576 (1.084, 2.292)	.017	1.930 (1.268, 2.935)	.002	1.935 (1.264, 2.960)	.002	1.963 (1.265, 3.048)	.003
Female	5.25 mg/dL	1.762 (1.212, 2.562)	.003	1.595 (1.043, 2.438)	.031	1.371 (0.871, 2.157)	.173	1.308 (0.821, 2.085)	.258

*Note:* Serum uric acid cutoff values: male (6.35 mg/dL); female (5.25 mg/dL). Model 1: unadjusted. Model 2: adjusted by (race, age, BMI). Model 3: adjusted by Model 2 + (LDL‐ cholesterol, fasting plasma glucose, serum creatinine). Model 4: adjusted by Model 3 + (angina/angina pectoris history, stroke history, smoking history, congestive heart failure history).

Abbreviation: CI, confidence intervals; OR, odds ratio; SUA, serum uric acid.

### 3.5 Discussion

In the US adult population, the aim of the present study was to investigate the association between SUA level and AAC. We provided the evidences that SUA level revealed a positive correlation with AAC score in a dose‐response manner. To the best of our knowledge, ours is the first study that evaluated the relationship between SUA and AAC.

Emerging evidences addressed that AAC was a recognized predictor of cardiovascular mortality.^6^, ^7^ Wilson PWF et al reported AAC deposits are a marker of subclinical atherosclerotic disease and an independent predictor of subsequent vascular morbidity and mortality in a 23‐year follow‐up study.^7^ An article published in the Nephrology Dialysis Transplantation in 2019 reported the relationship between dietary zinc intake and AAC in a national survey of US.^10^ Which demonstrated increased intake of dietary zinc was independently related to decreased odds of possessing severe AAC. In a similar way, determined mainly by dietary intake, elevated SUA level has been associated with conventional CVD risk factors including metabolic syndrome, dyslipidemia, obesity, hypertension, diabetes, and nonalcoholic fatty liver disease.^11^

No research has investigated the direct relationship between SUA and AAC in humans before. Notably, several researches have been established to substantiate an association between coronary calcification and SUA level.^12^, ^13^ Krishnan et al^12^ demonstrated that hyperuricemia is an independent risk factor for subclinical atherosclerosis in young adults by means of analyzing data of 2498 participants derived from the Coronary Artery Risk Development in Young Adults (CARDIA) study.^14^

In the same paper, Kiss et al illustrated that SUA level was associated with calcium scores and the third SUA tercile was an independent predictor for the presence of high‐risk coronary calcification. In contrast to the above study, we investigated not only the subsistence of overall AAC through four different AAC scores (AAC total 24 score, AAC anterior 8 score, AAC posterior 8 score and AAC total 8 score), but we also utilized the cutoff value of 2 for AAC total 24 score which defines subclinical atherosclerosis and found a strong and independent association in the male participants. The Framingham study also reported that gout had a significant effect on the preceding coronary events in male but not in female.^15^ This gender difference may be due to the action of the hormone estrogen.^16^ Estrogens have an impact on the renal tubular handling of uric acid and premenopausal levels of estrogen in women cause a greater renal clearance of uric acid.^17^ Little has been known why estrogen increases renal uric acid excretion. However, recently it has been revealed that estradiol suppressed the protein levels of urate reabsorptive transporters urate transporter 1 and glucose transporter 9 (Urat1 and Glut9), and that of urate efflux transporter ATP‐binding cassette subfamily G member 2 (Abcg2).^18^

Prior research has proven elevated SUA level to be an independent risk factor of atherosclerosis.^19^ There are several possible pathophysiological mechanisms linking SUA to atherosclerosis. First, SUA may result in the activation of local platelet aggregation and lysis,^20^, ^21^ which are related to an elevated risk of vulnerable plaque formation. Second, during the production of uric acid catalyzed by xanthine‐oxidase (XO), reactive oxygen species (ROS) are generated as by product, which have a significant role in the increased vascular oxidative stress.^22^ Third, SUA could also bring about stimulation of inflammatory pathways in vascular smooth muscle cell (VSMC) proliferation contributing to dysfunction of endothelium.^23^ The above‐mentioned alterations could take an important part in illustrating the possible pathophysiological mechanism between SUA and atherosclerosis in the abdominal aorta.

Several limitations still existed in this study. First, as the initial research in humans to investigate the association between SUA and AAC. Second, AAC data derived from NHANES were acquired through only participants 40 years of age and older; therefore, patients with lowest SUA level are excluded from this research. Third, we did not have data on frequency/precise amount of physical exertion, dietary intake, or medication, all of which can alter SUA level.

### 3.6 Conclusions

Our results highlighted the promising evidences that SUA level demonstrated a positive correlation with AAC score in a dose‐response manner. These findings decisively indicated that SUA may act as a promising tool to forecast the incidence of subclinical atherosclerosis in males.

## CONFLICT OF INTEREST

The authors declare no potential conflict of interest

## AUTHOR CONTRIBUTIONS

Yen‐Wei Li contributed to the design of the study, was responsible for the management and retrieval of data, contributed to initial data‐analysis and interpretation, and drafted the initial manuscript. Yen‐Wei Li and Wei‐Liang Chen decided upon the data collection methods. Yen‐Wei Li and Wei‐Liang Chen were also responsible for the data‐analysis decisions. Wei‐Liang Chen conceptualized and designed the study, supervised all aspects of the study, critically reviewed and revised the manuscript, and approved the final manuscript as submitted. All authors meet the ICMJE criteria for authorship.

## ETHICS STATEMENT

IRB information: NCHS IRB granted an exemption from requiring ethics approval.

## Supporting information


**Supplementary Table 1** Definitions of abdominal aortic‐calcification score.
Supplementary Table 2. Correlation table
Supplementary Table 3. Association between SUA (as quartiles comparison) and presence of subclinical atherosclerosis (high AAC score).
Supplementary Table 4. Optimal SUA cutoff values for predicting the presence of subclinical atherosclerosis in different gender groups.
