# Supplementary material for: Clinical relevance of serum uric acid and abdominal aortic‐calcification in a national survey
Source: Clin Cardiol. 2020 Jul 28;43(10):1194–201. doi: 10.1002/clc.23433 (PMC7533967; doi:10.1002/clc.23433)
Supplement: Supplementary file 1 — Supplementary Table 1 Definitions of abdominal aortic‐calcification score. Supplementary Table 2. Correlation table Supplementary Table 3. Association between SUA (as quartiles comparison) and presence of subclinical atherosclerosis (high AAC score). Supplementary Table 4. Optimal SUA cutoff values for predicting the presence of subclinical atherosclerosis in different gender groups. [file CLC-43-1194-s001.docx]

**Supplementary Table 1. Definitions of abdominal aortic calcification score.**

| Subject | Definition |
| --- | --- |
| AAC Total 24 Score | The anterior and posterior aortic walls are divided into four segments, resulting in a calcification score ranging from “0” to “6” for each segment and “0” to “24” for the total score. |
| AAC Anterior 8 Score | Ranging from “0” to “4” to evaluate the entire length of calcification of the anterior aortic walls. |
| AAC Posterior 8 Score | Ranging from “0” to “4” to evaluate the entire length of calcification of the posterior aortic walls. |
| AAC Total 8 Score | The sum of AAC Anterior 8 Score and AAC Posterior 8 Score. |

**Supplementary Table 2. Correlation table**

| **Variables** | 1 | 2 | 3 | 4 | 5 | 6 | 7 | 8 | 9 | 10 | 11 | 12 | 13 |
| --- | --- | --- | --- | --- | --- | --- | --- | --- | --- | --- | --- | --- | --- |
| **1.Serun uric acid** | 1 |  |  |  |  |  |  |  |  |  |  |  |  |
| **2.AAC Total 24 Score** | .077^**^ | 1 |  |  |  |  |  |  |  |  |  |  |  |
| **3.Age** | .132^**^ | .400^**^ | 1 |  |  |  |  |  |  |  |  |  |  |
| **4.BMI** | .214^**^ | -.091^**^ | -.054^**^ | 1 |  |  |  |  |  |  |  |  |  |
| **5.LDL-C** | .026 | -.096^**^ | -.160^**^ | .006 | 1 |  |  |  |  |  |  |  |  |
| **6.Glucose** | -.012 | .085^**^ | .068^**^ | .135^**^ | -.086^**^ | 1 |  |  |  |  |  |  |  |
| **7.Creatinine** | .260^**^ | .100^**^ | .143^**^ | .007 | -.081^**^ | .015 | 1 |  |  |  |  |  |  |
| **8.Gender** | -.353^**^ | -.004 | -.018 | .062^**^ | .073^**^ | -.049^*^ | -.260^**^ | 1 |  |  |  |  |  |
| **9.Race (Non-Hispanic White)** | .101^**^ | .013 | -.001 | -.165^**^ | -.038 | -.060^**^ | .069^**^ | -.016 | 1 |  |  |  |  |
| **10.CHF history** | -.033 | -.019 | -.015 | -.031 | .017 | .026 | -.044^*^ | -.013 | .006 | 1 |  |  |  |
| **11.Angina/Angina pectoris history** | -.012 | -.052^**^ | -.019 | -.009 | .032 | -.011 | -.031 | .023 | .013 | .325^**^ | 1 |  |  |
| **12.Stroke history** | -.015 | -.090^**^ | -.086^**^ | .012 | .095^**^ | -.037 | -.068^**^ | .000 | -.045^*^ | .174^**^ | .186^**^ | 1 |  |
| **13.Smoking history** | -.048^*^ | -.093^**^ | -.043^*^ | -.003 | .033 | -.013 | -.031 | .156^**^ | .037^*^ | -.013 | .007 | .002 | 1 |

*Significant at 0.05 level

**Significant at 0.01 level

**Supplementary Table 3. Association between SUA (as quartiles comparison) and presence of subclinical atherosclerosis (high AAC score).**

|  | **SUA quartiles** | **Model 1** | | **Model 2** | | **Model 3** | | **Model 4** | |
| --- | --- | --- | --- | --- | --- | --- | --- | --- | --- |
|  |  | **OR**  **(95% CI)** | **P value** | **OR (95% CI)** | **P value** | **OR (95% CI)** | **P value** | **OR**  **(95% CI)** | **P value** |
| **Total** | **Q2 vs Q1** | 1.068  (0.715, 1.597) | 0.747 | 1.081  (0.698, 1.675) | 0.727 | 1.078  (0.692, 1.680) | 0.740 | 1.071  (0.684, 1.677) | 0.764 |
|  | **Q3 vs Q1** | 1.381  (0.953, 2.003) | 0.088 | 1.457  (0.947, 2.242) | 0.087 | 1.443  (0.932, 2.237) | 0.100 | 1.449  (0.929, 2.260) | 0.102 |
|  | **Q4 vs Q1** | 1.876  (1.298, 2.711) | 0.001 | 2.038  (1.303, 3.187) | 0.002 | 1.935  (1.221, 3.065) | 0.005 | 1.956  (1.225, 3.124) | 0.005 |
| **Male** | **Q2 vs Q1** | 0.777  (0.354, 1.706) | 0.529 | 0.836  (0.359, 1.947) | 0.678 | 0.794  (0.338, 1.864) | 0.596 | 0.849  (0.359, 2.006) | 0.709 |
|  | **Q3 vs Q1** | 1.192  (0.604, 2.352) | 0.612 | 1.347  (0.644, 2.817) | 0.428 | 1.302  (0.619, 2.738) | 0.486 | 1.413  (0.667, 2.996) | 0.367 |
|  | **Q4 vs Q1** | 1.637  (0.839, 3.195) | 0.149 | 2.212  (1.052, 4.653) | 0.036 | 2.152  (1.015, 4.560) | 0.046 | 2.337  (1.091, 5.007) | 0.029 |
| **Female** | **Q2 vs Q1** | 1.232  (0.766, 1.979) | 0.390 | 1.187  (0709, 1.987) | 0.513 | 1.200  (0.707, 2.035) | 0.500 | 1.157  (0.674, 1.986) | 0.597 |
|  | **Q3 vs Q1** | 1.502  (0.902, 2.502) | 0.118 | 1.502  (0.852, 2.650) | 0.160 | 1.425  (0.788, 2.575) | 0.241 | 1.376  (0.746, 2.541) | 0.307 |
|  | **Q4 vs Q1** | 2.033  (1.176, 3.515) | 0.011 | 1.574  (0.838, 2.955) | 0.158 | 1.081  (0.536, 2.179) | 0.828 | 0.960  (0.464, 1.987) | 0.913 |

Subclinical atherosclerosis (high AAC score) defined as more than the 75th percentile of AAC Total 24 Score, cut-off point: 2

Model 1: unadjusted

Model 2: adjusted by (race, age, BMI, gender)

Model 3: adjusted by Model 2 + (LDL- cholesterol, fasting plasma glucose, serum creatinine)

Model 4: adjusted by Model 3 + (angina/angina pectoris history, stroke history, smoking history, congestive heart failure history)

**Supplementary Table 4. Optimal SUA cut-off values for predicting the presence of subclinical atherosclerosis in different gender groups.**

|  | Male | Female |
| --- | --- | --- |
| AUC (95%CI) | 0.516 (0.478, 0.555) | 0.577 (0.539, 0.616) |
| Sensitivity (%) | 38.4 | 47.5 |
| Specificity (%) | 66.2 | 68.0% |
| P-value | 0.398 | <0.001 |
| Cut-off value (mg/dL) | 6.35 | 5.25 |
